# When failure is not an option: a police firearms training concept for improving decision-making in shoot/don’t shoot scenarios

**DOI:** 10.3389/fpsyg.2024.1335892

**Published:** 2024-04-24

**Authors:** Joshua Olma, Christine Sutter, Sandra Sülzenbrück

**Affiliations:** ^1^Institute of Traffic and Engineering Psychology, German Police University, Münster, Germany; ^2^IWP Institute for Business Psychology, FOM University of Applied Sciences, Dortmund, Germany

**Keywords:** police, vision, attention, training, decision-making, shooting

## Abstract

Sometimes, policing requires a quick and correct assessment of potentially hazardous situations. The training of tactical gaze control and visual attention, and its positive impact on efficient shoot/don’t shoot decisions in police cadets’ use of firearms has recently been demonstrated. On this basis, we designed an individual videobased police firearms training that was grounded on the Four-Component Instructional Design Model (4C/ID). We shifted toward an individual blended learning approach where we applied an intervention training focused on situational awareness, tactical gaze control, and visual attention. In a preregistered lab experiment, *N* = 45 senior police officers were randomly allocated to the intervention training or an active control training that resembled a traditional police firearms training. Both groups watched a self-produced educational video before proceeding to the practical training in our indoor firing range. In a pre- and post-test, they engaged in realistic shoot/don’t shoot video scenarios. Both groups did very well regarding decision-making, the optimal muzzle position, and the tactical conduct to keep both eyes open before shooting. Although both groups performed on a comparable level in the pre-test’s shoot scenarios, the intervention group significantly improved their response times and time until the first hit. Overall, we were able to provide an adapted, didactically based police firearms training that supplements current standards. We demonstrated that experts are still susceptible to innovative training concepts and therefore substantiate the recommendation to devote more attention to approaches that emphasize the importance of situational awareness, tactical gaze control, and visual attention in police firearms training.

## 1 Introduction

### 1.1 Situational awareness and threat assessment in police work

Police officers can find themselves in situations that are potentially life-threatening all of a sudden. Therefore, police officers are expected to show situational awareness, assess threats correctly, and thus react adequately under stress at any time (Vickers, 1996; Helsen and Starkes, 1999; Martaindale, 2020). Even if fatal encounters are the exception, police officers must always be prepared to make use of their firearm. Shooting involves a series of complex actions, enabled only by high cognitive efforts (Biggs et al., 2015). Visual search is essential to find an appropriate target, whether to shoot or not premises decision-making, and squeezing the trigger involves response execution. These saccades of action rely on information for which visual perception proves to be the most important source (Körber, 2016; Heusler and Sutter, 2020a). Human visual perception is a widely researched field. In adult humans, more than one-third of the brain is involved in visual perception (Findlay and Gilchrist, 2003), thus playing a vital role for humans in interacting with their environment (Groner and Groner, 1989; Gilchrist and Harvey, 2006). Hence, visual perception facilitates, mostly even dominates the control of motor movements, the estimation of distances, and the use of tools (*cf.* Sutter and Ladwig, 2012; Ladwig et al., 2013; Sutter et al., 2013). Basic requirement for such cognitive efforts is a flow of information that can be both bottom-up (i.e., light rays on sensory receptors being the starting point for an upward process that leads to the formation of an internal description of the environment) and top-down (i.e., prior knowledge guiding the perceptual process).

Nevertheless, visual perception is also subject to certain limiting factors such as poor night vision, suppression of vision between saccades, and susceptibility to optical illusions (*cf.* Coren and Girgus, 1978; Findlay and Gilchrist, 2003; Rayner, 2009; Todorović, 2020). Rensink et al. (1997) were able to demonstrate that even obvious changes in the visual field can go unnoticed if the focus of attention is directed elsewhere (the so called “change-blindness paradigm”). It can therefore be concluded that most if not all visual control also requires attention (Wolfe, 2000). According to the “Posner paradigm” (Posner, 1980), this export is facilitated when attention is focused on only one area at a time. The larger the field of attention, the lower the density of resources available to the human information processing system (Eriksen and St. James, 1986). For this reason, the human brain tries to unconsciously shift the focus toward salient visual features that always attract attention, such as (happy) faces (Cerf et al., 2007; Calvo and Nummenmaa, 2008) or weapons (Biggs et al., 2013). Fostering factors that boost efficacy of vision include expertise, training, and a sound searching strategy (Henderson et al., 2007; Dewhurst and Crundall, 2008; Körber and Neuberger, 2009). Priming also proves beneficial for perceptual performance (Castelhano and Henderson, 2007; Körber, 2016).

As visual perception is the key element to police operations, it is not surprising that police officers are superior to non-police officers or novices in terms of gaze patterns, reaction times, marksmanship, and threat detection (Helsen and Starkes, 1999; Nieuwenhuys and Oudejans, 2010, 2011; Vickers and Lewinski, 2012; Renden et al., 2015, 2017; Körber, 2016). For instance, Heusler and Sutter (2020b) were able to show that experienced patrol officers rather focused on a suspect’s face whereas special force officers fixated the crucial hands and hip region. In another experiment by Taylor (2020), in which a lower muzzle position was taken (and thus allowing a view of the hands and hip region), the participating police officers significantly improved their decision to shoot or not to shoot without having to accept a significant loss of time. However, stress and anxiety have a negative effect on the performance of police officers, especially in situations that require the use of firearms (Nieuwenhuys and Oudejans, 2010, 2011; Nieuwenhuys et al., 2012; Renden et al., 2014, 2017; Landman et al., 2016; Donner et al., 2017). In a split second, police officers have to react correctly, and either protect uninvolved or unarmed persons from being harmed by police shooting, or act in self-defense when facing an attack. The correct decision-making poses a major challenge to the human information processing system, especially under elevated stress and anxiety levels. Blair et al. (2011) demonstrated in their experiment that police officers were not able to shoot at an armed suspect in time even if they already aimed their firearm at the attacker. Henriksen and Kruke (2020) observed that stressed police officers tended to hold their fire in threatening situations thus leading to an imminent attack which might have been avoidable if they had engaged earlier. At the same time, Witt and Brockmole (2012) showed that participants carrying a handgun are more likely to classify non-hazardous objects held by suspects as handguns. Consequently, the opportunity to use a handgun influences the (mis-) perception of handguns, thus leading to unjustified threat-induced behavior. Therefore, basing a shoot/don’t shoot decision on a profound threat assessment is vital, just as the ability to suppress a shooting impulse (so-called inhibitory control; Biggs and Pettijohn, 2022).

### 1.2 Specified firearm trainings and conceptual framework

To optimally prepare police officers for situations in which they may or may not have to use their firearms, they must know about what to expect (situational awareness), what to focus on (tactical gaze control and visual attention), and how to guide their decisions (decision-making). Perceptual performance and tactical gaze control significantly improve with just one or a few training sessions (Helsen and Starkes, 1999; Nieuwenhuys and Oudejans, 2011; Heusler and Sutter, 2022). In line with previous studies (*cf.* McCraty and Atkinson, 2012; Neuberger, 2013; Biggs et al., 2015, 2022; Hamilton et al., 2019; Preddy et al., 2019; Biggs and Pettijohn, 2022), Heusler and Sutter (2022) showed that their cognitive training can improve police cadets’ performance in shoot/don’t shoot scenarios. In general, cognitive training is intended to improve broad-ranging cognitive skills such as the aforementioned perception (visual, auditive, etc.), attention, memory, and decision-making. It is important to distinguish between general cognitive enhancement programs and more specific, task oriented cognitive training approaches. The former is often referred to as “brain training” that lacks ecological validity and applicability in real life (Renshaw et al., 2019). Key features to effective task oriented cognitive training are its representativeness and fidelity of (future) performance environments (Travassos et al., 2013). In law enforcement, adhering to those key features in training is particularly important because each decision in real life might have severe consequences. To achieve improvement through cognitive training, a considerable amount of approaches has been examined (for an overview see Simons et al., 2016; Blacker et al., 2019).

Helsen and Starkes (1999) pioneered the impact of slide and video simulation training on police officers’ decision-making in live fire scenarios. The learning tasks were object identification, decision-making, and shooting performance, eventually. During the training, the task difficulty gradually increased. Additionally, the authors measured eye-movements using a semi-mobile eye-tracking device. After training, the experimental group increased its performance to a higher degree than the control groups.

A few years later, Saus et al. (2006) investigated the effect of situational awareness training in a shooting simulator. Police students were armed with a gas-operated submachine gun and had to engage in three training tasks which required the decision to shoot/not shoot and a report on questions related to situational awareness. After training, the experimental group showed higher subjective and observer ratings of situational awareness than the control group.

To assess the benefits of reality-based training on high-pressure shooting performance, Oudejans (2008) had police officers engage in force-on-force roleplay with an opponent that shot back with FX ammunition (color cartridges). In three training sessions, the participants’ task was to defend themselves as realistically as possible against a knife and later handgun attack using their own FX gun. After training, the experimental group’s performance did no longer deteriorate whereas the control group’s performance was still poor. A later study was able to replicate this effect in a more realistic training setting (i.e., a police vehicle, a building, a street) and with a supplemental mobile eye-tracking device that allowed for gaze analysis (Nieuwenhuys and Oudejans, 2011).

In another approach, McCraty and Atkinson (2012) explored whether a resilience training can reduce physiological and psychological stress in police officers. Contrary to the previously mentioned studies, the training task did not comprise practical shooting but aimed at self-regulating the participants’ mental, emotional, and physical systems. The resilience training was given classroom-based and its effect was tested in three realistic force-on-force scenarios that included the use of FX ammunition. After training, the experimental group experienced reductions in physiological and psychological stress as compared to a control group.

Biggs et al. (2015) offered either response inhibition training or visual search training to civilian participants to assess the respective effect on performance in a simulated shooting environment. All participants completed a pre-training session with computer-based tasks (e.g., go/no-go task, Stroop task) before they were either trained to withhold a response and avoid certain stimuli (inhibition training) or trained to consistently perform a visual search task. Both trainings consisted of several sessions and the learning tasks gradually increased in difficulty. After training, the experimental group that received the response inhibition training reduced simulated civilian casualties to a higher extent than the control group.

To address the lack of either the participants’ expertise (police students and civilians) or the settings’ realism (force-on-force roleplay and simulated environments), Hamilton et al. (2019) created a training that was intended to improve experienced police officers’ performance in live fire exercises. On a daily basis, the participants completed three learning tasks over the course of 4 weeks. The learning tasks mainly aimed at visual processing speed, attention, and inhibitory control. After training, the experimental group performed better whereas the control group showed no improvement.

In another study, Biggs and Pettijohn (2022) tested the effect of a stress-inoculation training on perceptual judgments in force-on-force roleplays. To enhance the performance under elevated stress levels, military personnel engaged in close combat using FX ammunition. Weapon handling under direct fire was the main learning task. The effect of this practical training was then tested in different force-on-force roleplays. After training, the likelihood of firing at an unarmed person was reduced.

In a more sophisticated field study, Heusler and Sutter (2022) designed an intervention training as realistic as possible (by using live ammunition and dynamic video scenarios), which improved police cadets’ performance in shoot/don’t shoot live fire scenarios. In contrast to most of the previous studies, the authors focused on situational awareness, tactical gaze control, and visual attention. The authors found that in the post-test, the intervention group brought their handgun at eyesight level later and had both eyes open longer before shooting compared to an active control group. Furthermore, the intervention group achieved significantly better results regarding correct decisions in shoot scenarios and response time than the active control group. However, the control group improved their marksmanship skills to a greater extent than the intervention group.

The aforementioned studies (especially the findings of Heusler and Sutter, 2022) indicate that good decision-making is based on the optimized perception of visual information: Situational awareness (i.e., constantly monitoring the surrounding) is prerequisite to improved tactical gaze control and visual attention (i.e., shifting gaze to hands and hip region; detecting and focusing on potentially hazardous objects; if necessary, drawing the service weapon but maintaining a low muzzle position while keeping both eyes open). The latter facilitate the processing of critical stimuli (i.e., hazardous objects) which affects decision-making substantially. Generally, existing training approaches for law enforcement personnel differ significantly in terms of its underlying concepts about learning, theoretical and practical implementation, and the materials used. On the one hand, these approaches demonstrate that training under live fire conditions and using dynamic video scenarios or simulations provides the most realistic experience to the trainees (i.e., levels of stress, weapon handling, etc.). On the other hand, many if not most aspects of firearms training can be practiced with non-lethal weapons. Additionally, non-lethal weapons can evoke comparable feelings of stress and arousal like firing with live ammunition (*cf.* Staller and Zaiser, 2015) but in a much safer and more economical setting. The decisive factor is the application of non-static (i.e., moving) stimulus material in order to optimally reproduce the dynamism of a real police operation. Given those insights, situational awareness, tactical gaze control, and visual attention seem like necessary learning objectives for professional handling of the service weapon. Yet and to the best of our knowledge, we were not able to identify a realistic and dynamic law enforcement firearms training which imparted those learning objectives in a sound and established didactical framework.

Analogous to learning basic skills such as reading and writing, the use of service weapons should also be subject to a scientifically based curriculum. Lately, the *Four-Component Instructional Design Model* (4C/ID, for a detailed description, see van Merrienboer, 2020) receives a lot of attention and could serve as such a didactical framework. The 4C/ID derives from classical pedagogical education; however, the name indicates its origin: The model ties in with the tradition of instructional design, which originated in the military (Gagné et al., 1992; van Merrienboer and Dijkstra, 1997; van Merrienboer et al., 2002). According to the 4C/ID, an educational program consists of four components: (1) learning tasks, (2) supportive information, (3) procedural information, and (4) part-task practice. Learning tasks (1) are characterized by the integration of (non-routine and routine) skills, knowledge, and attitudes. They are holistic experiences which correspond to real-life tasks. Key features are variability (learning tasks must differ from each other), levels of complexity (increasing task demands), and scaffolding (decreasing support and guidance). The learning tasks aim at inductive learning. Supportive information (2) refers to the classic “theory” that supports the learning and performance of non-routine aspects of the learning task. It typically shows how a domain is organized and how to approach the task in this domain. Supportive information can be provided before or during a task and aims at elaboration. Procedural information (3) can also be called “just in time information” as it is usually given during the task. It is intended to help performing the routine aspects of a learning task. The procedural information aims at rule formation. Part-task practice (4) serves as additional practice for routine aspects that enable automaticity through increased repetition; it aims at strengthening of the rules. According to the 4C/ID, the four components are all linked in an integrated curriculum that enables the transfer of learning. A meta-analysis by Costa et al. (2022) concluded that the 4C/ID had a high impact on performance in various use cases (in particular higher education). Hence, it appeared reasonable that law enforcement firearms training could be based on this model. However, we did not identify any studies describing the use of the 4C/ID in this context. While research on law enforcement firearms training sometimes (and possibly rather accidently) made use of single components of the 4C/ID, a holistic approach is pending.

For the present study, we designed an individual video-based intervention training that was based on the 4C/ID and inspired by the field study of Heusler and Sutter (2022). Our research aim was to verify this intervention training’s efficacy on shoot/don’t shoot performance in a lab experiment. In line with the aforementioned study, we assumed that the intervention training will improve the participants’ efforts to optimize their perception of visual information. To do so, we hypothesized that the intervention group will bring their handgun up at eyesight level later before shooting than the control group (hypothesis 1). Furthermore, we hypothesized that the intervention group will keep both eyes open longer right before shooting than the control group (hypothesis 2). Both aspects will prevent from limiting one’s field of view, for instance by blocking the view of the crucial hands and hip region with the own hands and firearm. We further assumed that the optimized perception of visual information will have a positive impact on decision-making, and, in shoot scenarios, on response preparation and response execution. We hypothesized that the intervention group will improve their number of correct decisions to shoot in shoot scenarios and to not shoot in don’t shoot scenarios to a greater extent than the control group (hypothesis 3). Concerning response preparation and response execution in correctly detected shoot scenarios, we hypothesized that the intervention group will shoot faster than the control group (hypothesis 4). We added a manipulation check as a baseline measurement for marksmanship: Matching the focus of the control training, we hypothesized that the control group will improve their traditional marksmanship skills more than the intervention group (hypothesis 5).

## 2 Materials and methods

### 2.1 Design

Our study design was a randomized controlled trial in a 2 (group) × 2 (measurement time) design. The intervention group received the specified firearms training while the control group was given an active control training that was in line with the common German police standard (see section 2.4). Both training concepts included a theoretical and a practical part. In the pre- and post-test, we measured the shooting performance of both groups (see section 2.3). Our dependent variables are described in section 2.6.

The study was conducted under the Declaration of Helsinki and the Ethical Principles and Protocol Code of the German Association of Psychologists (adapted from the American Psychological Association (APA) Code of Ethics). The Ethics Committee of the German Police University (“*Ethik-Kommission der Deutschen Hochschule der Polizei”*) provided approval of the study (approval number: “DHPol-EthK.2022.Olm3”). We preregistered the study on www.aspredicted.org (#119896).

### 2.2 Participants

Via notices on the campus, we recruited *N* = 45 senior police officers (female = 14; male = 31; mean age = 35.29, *SD* = 5.01, *min* = 27, *max* = 44) for our study. On average, the participants had been in the police force for 12.98 years (*SD* = 7.21, *min* = 1, *max* = 24). All senior police officers participated voluntarily and were able to abort the study at any time. At the end of the study, they were debriefed. All participants gave their informed consent for inclusion before they participated in the study. We were not able to include wearers of glasses (see section 2.3).

A total of *n* = 23 participants was randomly assigned to the control group whereas *n* = 22 participants received the intervention training. Our sample size was in line with comparable studies (Helsen and Starkes, 1999; Nieuwenhuys et al., 2012; Vickers and Lewinski, 2012; Heusler and Sutter, 2020b, 2022; Tawa et al., 2022). We ascertained the participants’ *Years of Service*, their standard service *Handgun*, their *Gender*, and their *Age* as demographic variables. To rule out possible bias due to confounding variables, we examined those demographic variables more thoroughly: *Years of Service*: Both groups did not differ significantly from each other in terms of mean years of service [mean years of service control grou*p* = 12.78, *SD* = 7.9; mean years of service intervention group = 13.18, *SD* = 6.59; *t*(43) = 0.18, *p* = 0.855].*Handgun*: In the intervention group, *n* = 9 participants also used the same model of handgun utilized during our training on duty, while *n* = 13 participants used different models in their police departments. The control group did not differ significantly with regard to this ratio [ours = 8, other = 15; χ^2^(1, *N* = 45) = 0.01, *p* = 0.908].*Gender*: Although we had more male than female participants, the distribution among the groups was balanced [control group: female = 7, male = 16; intervention group: female = 7, male = 15; χ^2^(1, *N* = 45) < 0.01, *p* = 1].*Age*: Both groups did not differ with regard to mean age (control group: mean age = 34.65, *SD* = 5.32; intervention group: mean age = 35.95, *SD* = 4.69; *W* = 1012.50, *p* = 1).Since both groups did not differ significantly in the expression of *Years of Service*, *Handgun, Gender*, and *Age*, we assumed that our results were not biased by demographic characteristics.

### 2.3 Pre- and post-test

**Figure 1:**
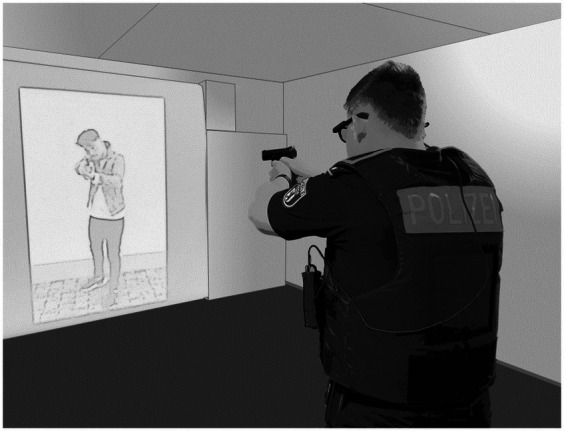
Experimental setup.

For shooting, participants used a gas-operated replica of a Heckler and Koch SFP9 handgun (in some countries: Heckler and Koch VP9) and an appropriate holster. This handgun is the standard service weapon in six German federal states [which represent about 43.00% of all police officers in Germany; Federal Statistical Office of Germany (Destatis), 2021]. In terms of characteristics, weight, and processed materials, this replica barely differs from the original, except for the ammunition. The handgun operates on small airsoft bullets, so-called *BBs* (*baby bullets*). The BBs are powered by gas that is filled into the magazine base. The gas allows semi-automatic use of the handgun, including recoil and bolt catch, once the magazine is empty. If the magazine is not inserted in the handgun, it cannot be fired. Albeit the gas handgun we used is an economic and non-lethal alternative, this weapon also posed a risk of injury, which we countered with in-depth briefing on the correct handling prior to the first task. The indoor firing range was set up in a lab room (5.50 m by 4.50 m) at the German Police University (see Figure 1). At one end of the rectangular room, a white paper canvas (2.00 m width by 2.20 m height) was attached to a roll that rested on top of two large cabinets. The cabinets were wrapped in white paper uniformly to the paper canvas. The area behind the paper canvas was lined with polystyrene and served as a bullet trap. To record the shooting performance, we set up a night vision camera (TP-Link Tapo C200) behind the paper canvas. Participants were asked to take position behind a marking 4.50 meters opposite the paper canvas. The targets were presented on the canvas with an Epson 2247 U projector (video and sound presentation) controlled by an experimental computer (Dell Latitude 5410).

For eye-tracking, we used the mobile and calibration-free eye-tracking system “Pupil Invisible” by Pupil Labs. Barely bigger and heavier than ordinary glasses, Pupil Invisible glasses can record eyelid movements at 200 Hz (infrared LEDs; resolution: 192 × 192 pixels) and create first-person videos at 30 Hz with a detachable front camera (resolution: 1,088 × 1,088 pixels). The eye-tracking system also has a microphone and is directly linked to a OnePlus 6 smartphone that the participant stowed in a pocket. During the pre- and post-test, the Pupil Invisible glasses served as eye protection. Use for wearers of ordinary glasses was not possible.

The pre- and post-test was divided into two tasks. Task 1 tested classic marksmanship skills, such as those required for regular shooting requalification by German law enforcement agencies. Our participants were instructed to first fire at two larger red circles (⌀ ~ 14 inches) and then at two smaller blue circles (⌀ ~ 7 inches), each once and as quickly and accurately as possible. They were free to choose on which of the large red circles they fired first. However, the shot was not repeated in case of a miss. Task 1 served as both a baseline and manipulation check insofar as the effect of both trainings on classical marksmanship was to be evaluated.

Task 2 confronted the participants with three realistic video scenarios that tested their shoot/don’t shoot performance. Prior to video onset, the participants were instructed that they had to tactically work through three scenarios autonomously, all of which were based on the same initial situation: A few hours after an armed robbery at a kiosk, the participant, together with his fictitious colleague, encounters a person who fits the description of the suspect. The suspect had a criminal record, was possibly armed, and unpredictable. A current mugshot was shown. The participants were encouraged to act as realistic as possible including movements, communication, and the free choice of the initial muzzle position. The firing of the service weapon in the event of an attack was explicitly endorsed while shooting at an unarmed suspect was seen as a clear mistake. The participants were shown six different video scenarios in total: Three in the pre-test and three in the post-test. Per measurement time, the first two scenarios were either shoot and don’t shoot or vice versa. The third scenario could be either but served as a dummy scenario, solely. This was to prevent participants from anticipating how many shoot and don’t shoot scenarios they would encounter. In total, we had 16 video scenarios (shoot = 8; don’t shoot = 8) that showed four different suspects: A woman and a man, both around 25 years old, and another woman and another man, both around 55 years old. All four wore the same outfit: Dark shoes, dark denim jeans, a white shirt, and a blue denim jacket. The background was a bright wall. For each suspect, we recorded two shoot and two don’t shoot videos whose content was the same across suspects^1^: In shoot scenario A, the suspect holds a smartphone in her/his left hand, telephoning, and does not react for a while. Both hands are well visible. Irritated, she/he asks what the spectator wants from her/him. After some time without any significant response, the suspect slowly reaches for her/his back pocket with the right hand and draws a black handgun that she/he points toward the spectator. In shoot scenario B, the suspect initially refuses to cooperate. Both hands are well visible. After a while, she/he pretends to do so but reaches for her/his left jacket pocket from which she/he retrieves a black handgun and points it toward the spectator. In don’t shoot scenario C, the suspect initially refuses to cooperate. Both hands are well visible. After some time, she/he reaches into her/his left jacket pocket and retrieves a dark leather wallet to present her/his ID in annoyance. In don’t shoot scenario D, the suspect initially refuses to cooperate. Both hands are well visible. After some time, she/he reaches into her/his back pocket and retrieves a black smartphone with which she/he intends to call a lawyer.

**Figure 2:**
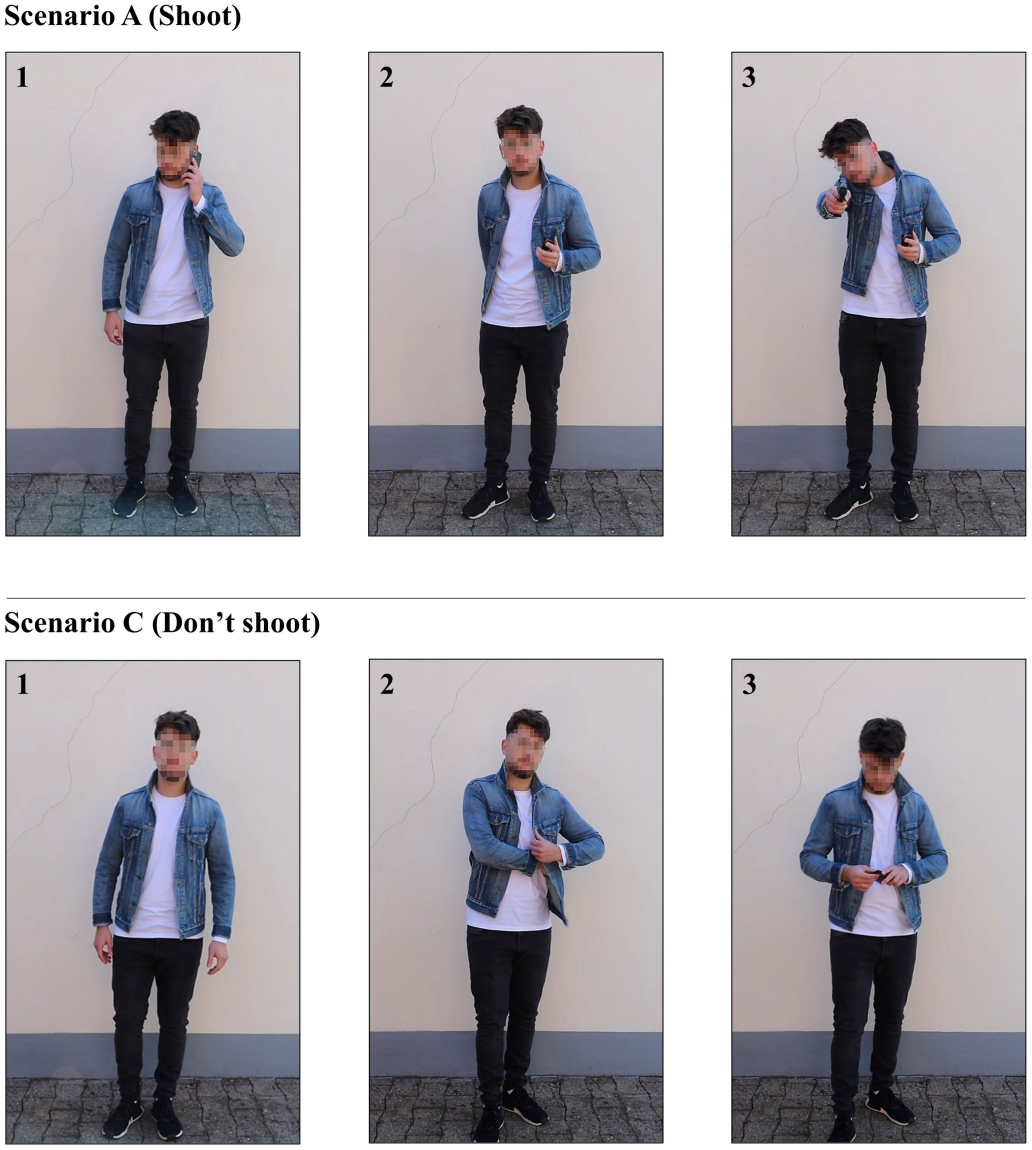
An exemplary shoot (A) and don’t shoot scenario (C). (1) Initial situation, (2) drawing motion begins, and (3) final position.

The video scenarios had a mean duration of 19 s (±5 s). The time of the motion sequences was approximately the same in all videos. The black handgun was clearly recognizable as such due to the contrast with the wall and the shirts of the suspects. The smartphone as well as the wallet could be clearly distinguished from the handgun. Both shoot and don’t shoot scenarios were designed in such a way that the outcome remained open until the end but the decision to shoot or not to shoot was unambiguous and left no room for hesitation. The black handgun was clearly identifiable for at least 2 s because in real life, this was sufficient time for both the officer and opponent to engage in lethal fire. Figure 2 depicts two exemplary video scenarios.

The video scenarios were specifically developed for the present study. They were recorded with a Canon EOS 750 D on a tripod, to which we attached a Sennheiser MK 400 microphone, and post-edited with DaVinci Resolve by Blackmagic Design. The distance between the camera and the suspect corresponded to the distance between the participant and the paper canvas/subject in the lab (4.50 m). We recorded at the eyesight level of a 1.75 m tall person, so that the suspects appeared on the paper canvas in their original size (1.65–1.90 m). We added subtle traffic sounds to increase the realism. All four actors volunteered and consented to the use of their video footage for our research purposes.

### 2.4 Training

**Figure 3:**
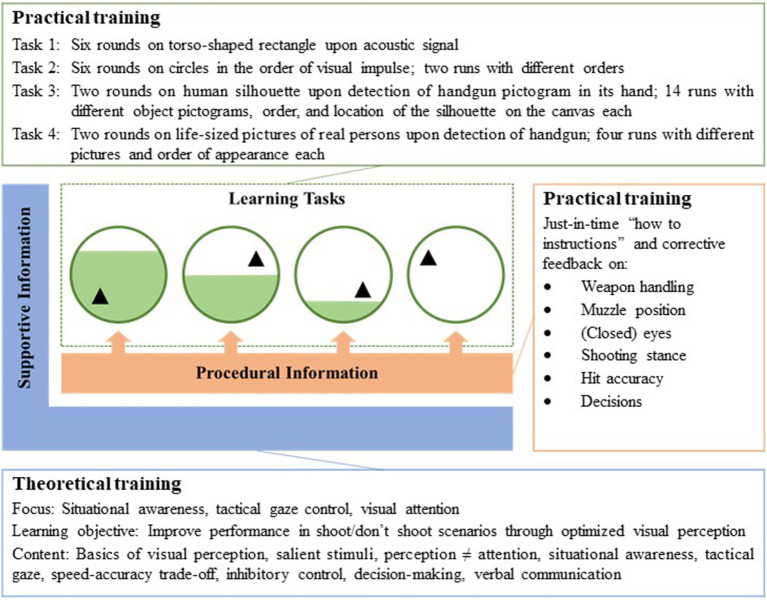
The intervention training, embedded in the 4C/ID (graphic based on van Merrienboer, 2020).

While the control training was similar to traditional police firearms training, the focus of the intervention training was on situational awareness, tactical gaze control, and visual attention. In addition, the intervention training was embedded in a modern didactic framework based on the 4C/ID, although we also applied some of its components in the control training. The training for both groups was divided into two parts: A theoretical training, that corresponded to the supportive information (component 2), and a practical training, that combined the learning tasks (component 1) and the procedural information (component 3). In our didactic framework, we omitted the part-task practice (component 4) for two reasons: First, a short lab experiment left little time for additional exercises and second, our learning tasks provided a sufficient amount of practice thus making additional exercises superfluous (*cf.* van Merrienboer, 2020). Figure 3 depicts the intervention training as we applied it to the intervention group.

For both groups, the theoretical training consisted of a self-produced educational video. With the digitization of teaching and the widespread availability of e-learning, educational videos are a state-of-the-art approach to effectively transfer knowledge to the spectator (Zhang et al., 2006; Poxleitner and Wetzel, 2014; Brame, 2016; Ebner and Schön, 2018). In our study, we were moreover able to ensure that all participants in a group were presented with the same content – at any time and with the push of a button. The educational videos lasted 12 min (control group) or 18 min (intervention group) and were shown in a separate room on a 25 inches monitor (AOC 24P1). The participants were provided with headphones (Logitech 960). According to the 4C/ID, the supportive information given through our educational videos was intended to help the participants connect existing knowledge and new input in order to successfully perform the subsequent learning tasks. The structure for both educational videos was the same: A hooded police firearms trainer in tactical gear welcomed the spectator and informed about the focus of the training and the learning objectives. Next was a theoretical block, which was followed by a summary. In line with the 4C/ID, the educational videos contained domain models that were illustrated by graphics and examples. The content of the educational video for the intervention group (see Figure 3) was carefully aligned with the learning objective: First, the spectator was introduced to the basics of human visual perception and its susceptibility to distortions. It was explained that some stimuli automatically draw attention (e.g., faces), and that perception and attention are two non-synonymous concepts. Then, the instructor explained what to expect and what to be aware of when entering certain police operational situations (situational awareness). It was outlined where to look at (hands and hip region) and how to distinguish hazardous from non-hazardous objects (visual attention). In preparation for a shoot/don’t shoot decision, the spectator was instructed to keep a muzzle position that allowed a clear view on the suspect as long as possible and to keep both eyes open when aiming at the suspect (tactical gaze control). Finally, the role of inhibitory control on decision-making and the effects of the speed-accuracy trade-off on response times and effective hits were elaborated. In addition, the spectator was encouraged to use verbal communication (both for clear instructions and de-escalation).

The educational video for the control training focused on the speed-accuracy trade-off of aiming and shooting, and its learning objective was to increase speed while maintaining accuracy. The theoretical input incorporated the process of shooting, the speed-accuracy trade-off, different shooting modes, the optimal use of sights, and the optimal movement of the handgun. For each educational video we created a so-called canvas before production (see Ebner et al., 2021). The videos were recorded in front of a green screen and then cut and post-edited with DaVinci Resolve by Blackmagic Design. This time, we used a Nikon D3200 camera and a Sennheiser ME 2 omnidirectional lavalier microphone. The prescribed text was read on a teleprompter. For optimal lighting, we used two studio lights on tripods.

The practical training took place in the indoor firing range (see Figure 1). For the exercises, the participants used the same handgun as in task 1 and task 2 in the pre- and post-test. The learning tasks for the intervention training (see Figure 3) were carefully designed according to the 4C/ID: The four exercises were meaningful whole tasks that gradually became more realistic, thus creating higher fidelity and variability (indicated by the small triangles in Figure 3). We followed the principle of scaffolding and gradually withdrew support and guidance (indicated by the filling of the circles in Figure 3). Furthermore, we set standards for acceptable performance in the learning tasks ex-ante for which we referred to the experiences from the field study of Heusler and Sutter (2022) as a benchmark. The participants were encouraged to apply the acquired supportive information from the educational video in the learning tasks. Constantly, they received procedural information, i.e., “how to instructions” and corrective feedback for improvement. Hereby, we intended not only to draw the participants’ attention to their errors but to explain the causes and give advice to resolve them. Whereas the first two tasks are intended to introduce the participants to only open fire after an acoustic signal or the visual search for targets, the latter tasks demand the participants to base their decision on actively distinguishing hazardous from harmless stimuli and thus learning to inhibit their impulse to fire.

In the control training, we also gave procedural information but rather neglected the design principles proposed by the 4C/ID. Participants had to shoot predefined targets that differed in shape, location, and size but always remained abstract stimuli. In the first task, they were instructed to shoot five rounds on a square upon an acoustic signal (two runs with squares of different sizes). Second, they had to shoot one round on each of four colored squares clockwise and a finishing round in a center circle upon an acoustic signal. Third, the participants shot five rounds on shrinking squares upon appearance on the canvas. The fourth task was equal to the second but the order of targets was now indicated by their color (two runs with different orders). The fifth and last task let the participants shoot five rounds on a bullseye target upon an acoustic signal.

### 2.5 Procedure

Data were collected in February and March 2023. The experiment lasted 50 min and was conducted by two researchers: The experimental supervisor (first author) monitored the process and an experienced, licensed police firearms trainer was responsible for the execution of the shooting tasks and the safe handling of the handgun by the participants. Since the theoretical training did not take place in the indoor firing range, we were able to test two participants simultaneously with a temporal offset. Depending on how many participants registered for a given day, either the experimental supervisor or police firearms trainer greeted the participants. The previous participant was then either still watching the educational video or was already doing the post-test.

All participants were introduced to the subject of the experiment and then asked for their written consent and demographic data. The objective of the experiment as well as the randomized assignment to one of the groups remained undisclosed. In preparation for the pre-test, the participants were given the safety briefing and either a service belt or a suitable holster. The police firearms trainer instructed them on the handgun’s specifics and encouraged them to engage in some dry practice to familiarize themselves with both the weapon and the holster. When the participants were ready, they were equipped with the mobile eye-tracking device and two full magazines (one for the magazine pouch, each 15 rounds) and were asked to take position behind the marking. The police firearms trainer started the recordings, positioned himself diagonally right next to the participants, and switched off the light. Now the participants were allowed to load the handgun. With a remote control, the police firearms trainer controlled the experimental presentations. Both pre- and post-test lasted 5 min. The two tasks were preceded by a written, detailed instruction while the target presentation was preceded by a 3 s countdown. After the pre-test, the light was switched on, the participants removed the magazines from the handgun, and returned the whole equipment. They were then sent to an adjacent room for the theoretical video-training. Meanwhile, the experimenters prepared the indoor firing range (disinfection of the equipment, reloading of the magazines, taping of the bullet holes with patches, and unrolling of the paper canvas) either for the subsequent participant (pre-test) or the current participant (practical training). During the practical training, participants were given a full magazine for each of the exercises. Following the practical training, the participants worked on the post-test tasks according to the sequence of the pre-test tasks. At the end of the experiment, the participants were informed about its objective and had the opportunity to give feedback. Again, we prepared the equipment and the indoor firing range for the next participant.

### 2.6 Dependent variables

In total, we measured six dependent variables: *Hit Factor* in task 1 (manipulation check) and *Decisions, Response Time*, *First Hit*, *Muzzle Position*, and *Closed Eye(s)* in task 2.

The *Hit Factor* in task 1 serves as a common indicator of marksmanship and is calculated by dividing the hits in four circles by the time taken (in seconds). Three hits in exactly 4 s would result in a *Hit Factor* equal to 0.75. However, the quotient can exceed 1.00 and the higher the value, the better the performance. In the present experiment, the *Hit Factor* was the central measure of task 1 and served to test hypothesis 5.

The variable *Decisions* in task 2 (shoot and don’t shoot scenarios) indicates the participants’ decision-making progress from pre- to post-test for shoot and don’t shoot scenarios separately. For the shoot scenarios, there were two possible outcomes: Did the participants shoot upon detection of the handgun, their decision was considered correct positive. Did they not shoot even though the black handgun was clearly visible for 2 s or did they shoot before the handgun was clearly identifiable, their decision was considered false negative. Analogously, holding back the fire in don’t shoot scenarios was considered correct negative and shooting at an unarmed suspect false positive. Each false decision was scored with “0,” each correct one with “1.” By subtracting the pre-test score from the post-test score, we calculated a value that indicated each participant’s progress (“–1” = deteriorated, “0” = constant, “1” = improved). The variable *Decisions* served to test hypothesis 3.

The *Response Time* in task 2 (shoot scenarios – correct positives only) provides information as a measure of the time (in milliseconds) between the detection of the black handgun and the participants’ initial motor response (shot taken on the paper canvas). Whether the participants hit the suspect with their shot was irrelevant. For the analysis, we time-stamped the moment when the black handgun was first clearly identifiable and measured the time until the first shot penetrated the paper canvas. The *Response Time* served to test hypothesis 4.

The *First Hit* in task 2 (shoot scenarios – correct positives only) describes the time (in milliseconds) between the detection of the black handgun and the first effective hit in predefined areas (the suspects’ torso or head). Its calculation is analog to *Response Time*. Did the participants not hit the predefined areas, their results were disregarded. The *First Hit* served no hypothesis because of the insignificant results of Heusler and Sutter (2022). Nevertheless, we kept this dependent variable in case our sample showed different results.

The *Muzzle Position* in task 2 (shoot scenarios – correct positives only) is a measure of the time (in milliseconds) that the participants held the handgun at eyesight level and pointed it at the suspect before taking the first shot. A tactical high ready position was not considered eyesight level. A minimized time is beneficial as the view on the attacker is not obscured by one’s hands and firearm. The *Muzzle Position* served to test hypothesis 1.

The *Closed Eye(s)* in task 2 (shoot scenarios – correct positives only) describes the time (in milliseconds) that the participants had at least one eye closed before taking the first shot. Again, a minimized time is beneficial because closing an eye limits one’s field of view. The *Closed Eye(s)* served to test hypothesis 2.

## 3 Results

### 3.1 Statistical analyses

Our statistical analyses were carried out using the software R. We chose α = 5% as our level of significance. For *Hit Factor*, *Response Time*, *First Hit*, *Muzzle Position*, and *Closed Eye(s)*, we calculated a repeated measures ANOVA with the within-subject factor *Time* and the between-subject factor *Group* for each dependent variable separately. Furthermore, and when reasonable, we determined the respective group’s progress for each dependent variable by calculating a separate repeated measures ANOVA with the factor *Time*, solely. For *Decisions*, we resorted to a non-parametric Wilcoxon Rank Sum Test.

For the *Hit Factor* analysis, we had to exclude 2 data sets due to a weapon malfunction. This left us with *N* = 43 evaluable data sets (control group = 21; intervention group = 22). For the *Response Time* analysis, we had to exclude the data sets of 7 participants that did not shoot in at least one of the shoot scenarios (false negatives). This left us with *N* = 38 evaluable data sets (control group = 21; intervention group = 17). For the *First Hit* analysis, we had to exclude the data sets of the same 7 false negatives and another 13 participants who did not hit the predefined areas in at least one of the shoot scenarios. This left us with *N* = 25 evaluable data sets (control group = 13; intervention group = 12). For the *Muzzle Position* analysis, we had to exclude the data sets of the 7 false negatives and another 14 participants who shot before bringing the handgun up at eyesight level. This left us with *N* = 24 evaluable data sets (control group = 12; intervention group = 12). For the *Closed Eye(s)* analysis, we had to exclude the data sets of the 7 false negatives and another 32 participants that kept both eyes open before taking the shot in the pre- and post-test. This left us with *N* = 6 evaluable data sets (control group = 5; intervention group = 1).

Additionally, we conducted exploratory analyses on the stimulus material. The participants were presented with different shoot scenarios in the pre- and post-test which is why we calculated an ANCOVA for each measurement time with the between-subject variable *Group,* the covariate *Suspect* and the respective outcome variable. Furthermore, we were interested in the shooting characteristics which is why we analyzed the number of shots fired (*Shots*), the number of hits (*Hits*), its *Percentage* and which of the fired shots hit first (*Ranking*) for both groups. We calculated a repeated measures ANOVA with the within-subject factor *Time* and the between-subject factor *Group* for *Shots* and *Hits*. For Ranking, we used a two-sided Fisher-test.

Both the analyses on the stimulus material and the shooting characteristics were limited to the shoot scenarios.

### 3.2 Manipulation check

For *Hit Factor*, the repeated measures ANOVA (*Time* × *Group*) showed a significant, medium effect for the factor *Time* [*F* (1,82) = 7.81, *p* = 0.006; η_p_^2^ = 0.09, 95% CI = [0.04, 0.29]] and a non-significant, small effect for the factor *Group* [*F*(1,82) = 0.31, *p* = 0.580; η_p_^2^ = 0.01, 95% CI = [−0.17, 0.16]]. An interaction of the factors *Time* × *Group* could not be shown [*F*(1,82) = 0.43, *p* = 0.512; η_p_^2^ = 0.01, 95% CI = [−0.16, 0.31]]. The overall performance improved from pre- to post-test (*M*_pre_ = 0.62, *SD*_pre_ = 0.30; *M*_post_ = 0.79, *SD*_post_ = 0.24; *min*_pre_ = 0, *max*_pre_ = 1.42; *min*_post_ = 0.30, *max*_post_ = 1.33).

### 3.3 Main results

For *Decisions*, both groups decided very well, and performance in shoot scenarios (mean progress of control group = 0.09, *SD* = 0.29; mean progress of intervention group = 0.18, *SD* = 0.39; *W* = 229, *p* = 0.365) and don’t shoot scenarios (mean progress of control group = 0; mean progress of intervention group = 0) did not differ significantly between pre- to post-test. Table 1 shows the ratio of correct positive and false negative decisions (shoot scenarios), and correct negative and false positive decisions (don’t shoot scenarios) for the control group (*n* = 23) and the intervention group (*n* = 22) per measurement time. In the shoot scenarios, the majority of participants made the correct decision in the pre-test. In the post-test, the only mistake was a false negative decision which means that the participant refrained from shooting. In the don’t shoot scenarios, all participants correctly decided to not shoot (correct negative) at both measurement times. Accordingly, we cannot report false positive decisions.

**Table 1:** Ratio of correct and false decisions for both groups.

	**Pre-test**		**Post-test**	
	Control group	Intervention group	Control group	Intervention group
Correct positive	21	17	23	21
False negative	2	5	0	1
Correct negative	23	22	23	22
False positive	0	0	0	0

**Figure 4:**
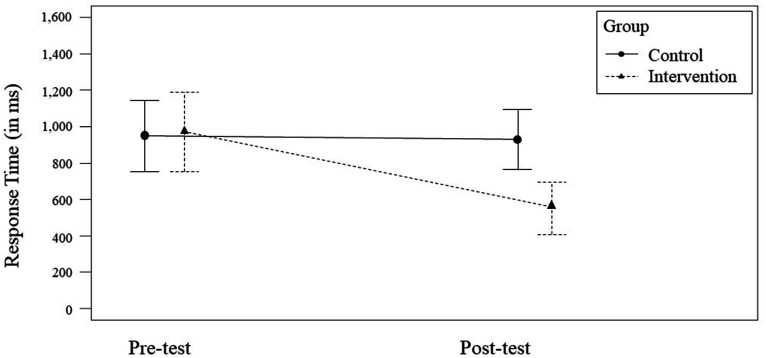
Significant *Time* × *Group* interaction effect for *Response Time*. Error bars represent the 95% CI for the mean.

For *Response Time*, we observed a non-significant, medium effect for the factor *Time* [*F*(1,72) = 5.01, *p* = 0.028; η_p_^2^ = 0.07, 95% CI = [−0.25, 0.22]] and a non-significant, small effect for the factor *Group* [*F*(1,72) = 4.12, *p* = 0.046; η_p_^2^ = 0.05, 95% CI = [−0.23, 0.27]]. The interaction of the factors *Time* × *Group* was significant with a medium effect size [*F*(1,70) = 5.14, *p* = 0.026; η_p_^2^ = 0.07, 95% CI = [−0.75, −0.05]] (see Figure 4). When considering the training groups separately, the control group (*n* = 21) did not significantly improve their performance from the pre- to post-test ([*F*(1,40) = 0.02, *p* = 0.889; η_p_^2^ < 0.01, 95% CI = [−0.27, 0.23]]; *M*_pre_ = 948 ms, *SD*_pre_ = 434; *M*_post_ = 931 ms, *SD*_post_ = 365), whereas the intervention group (*n* = 17) significantly improved its *Response Time* ([*F*(1,32) = 11.59, *p* = 0.002; η_p_^2^ = 0.27, 95% CI = [−0.75, −0.05]]; *M*_pre_ = 969 ms, *SD*_pre_ = 421; *M*_post_ = 551 ms, *SD*_post_ = 280).

**Figure 5:**
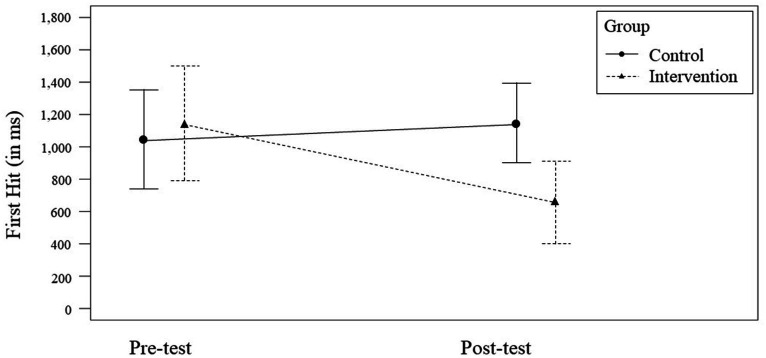
Significant *Time* × *Group* interaction effect for *First Hit*. Error bars represent the 95% CI for the mean.

For *First Hit*, we observed a non-significant, small effect for the factor *Time* [*F*(1,46) = 1.86, *p* = 0.179; η_p_^2^ = 0.04, 95% CI = [−0.27, 0.47]] and a non-significant, small effect for the factor *Group* [*F*(1,46) = 2.17, *p* = 0.148; η_p_^2^ = 0.05, 95% CI = [−0.28, 0.48]]. The interaction of the factors *Time* × *Group* was significant with a medium effect size [*F*(1,46) = 4.90, *p* = 0.032; η_p_^2^ = 0.10, 95% CI = [−1.13, −0.05]] (see Figure 5). When considering the respective group, the control group (*n* = 13) did not significantly improve their performance from the pre- to post-test ([*F*(1,24) = 0.32, *p* = 0.578; η_p_^2^ = 0.01, 95% CI = [−0.27, 0.47]]; *M*_pre_ = 1,044 ms, *SD*_pre_ = 506; *M*_post_ = 1,146 ms, *SD*_post_ = 406) whereas the intervention group (*n* = 12) significantly improved its *First Hit* ([*F*(1,29) = 6.11, *p* = 0.022; η_p_^2^ = 0.22, 95% CI = [−0.90, −0.08]]; *M*_pre_ = 1,143 ms, *SD*_pre_ = 555; *M*_post_ = 654 ms, *SD*_post_ = 401).

For *Muzzle Position*, we observed a non-significant, small effect for the factor *Time* [*F*(1,44) = 0.15, *p* = 0.705; η_p_^2^ < 0.01, 95% CI = [−1.66, 2.59]] and a non-significant, small effect for the factor *Group* [*F*(1,44) = 0.13, *p* = 0.716; η_p_^2^ < 0.01, 95% CI = [−1.11, 3.15]]. The interaction of the factors *Time* × *Group* was non-significant with a small effect size [*F*(1,44) = 1.00, *p* = 0.322; η_p_^2^ = 0.02, 95% CI = [−4.50, 1.51]]. Table 2 shows that the control group (*n* = 12) increased the time of having the handgun at eyesight level before shooting whereas the intervention group (*n* = 12) decreased that time.

**Table 2:** Mean *Muzzle Position* (in ms) for both groups for pre- and post-test.

	**Pre-test**	**Post-test**
Control group	786 (*SD* = 1,256)	1,249 (*SD* = 3,222)
Intervention group	1,806 (*SD* = 3,679)	744 (*SD* = 1,104)

For *Closed Eye(s)*, 32 participants correctly kept both eyes open before shooting. Table 3 shows that the control group (*n* = 5) increased the time of having at least one eye closed before shooting whereas the intervention group (*n* = 1) slightly decreased the time. Meaningful inferential statistical results were not expected because of the small group size, so we did not continue the analysis of *Closed Eye(s)*.

**Table 3:** Mean *Closed Eye(s)* (in ms) for both groups for pre- and post-test.

	**Pre-test**	**Post-test**
Control group	382 (*SD* = 236)	458 (*SD* = 258)
Intervention group	213	197

### 3.4 Exploratory analyses

To begin with, we were interested if the handgun utilized during our training had an effect on the participants’ performance. However, we discovered that there were no significant interaction effects with *Handgun* as a covariate and that the pattern of results did not substantially change with the addition of the covariate.

Furthermore, we wanted to explore whether the type of stimulus material shown had an effect on the performance of the participants. For the shoot scenarios, we had four different suspects with two different versions each (see section 2.3). The four different suspects were presented randomized but counterbalanced across the two groups and per measurement time [pre-test: χ^2^(3, *N* = 45) = 1.28, *p* = 0.733; post-test: χ^2^(3, *N* = 45) = 1.48, *p* = 0.687; see Table 4].

**Table 4:** Presentation of the video scenarios.

	**Pre-test**		**Post-test**	
	Control group	Intervention group	Control group	Intervention group
Suspect 1 (Young female)	Total = 4 (*A* = 2, *B* = 2)	Total = 7 (*A* = 4, *B* = 3)	Total = 6 (*A* = 2, *B* = 4)	Total = 3 (*A* = 2, *B* = 1)
Suspect 2 (Young male)	Total = 8 (*A* = 4, *B* = 4)	Total = 6 (*A* = 4, *B* = 2)	Total = 5 (*A* = 2, *B* = 3)	Total = 7 (*A* = 4, *B* = 3)
Suspect 3 (Older female)	Total = 6 (*A* = 3, *B* = 3)	Total = 5 (*A* = 4, *B* = 1)	Total = 7 (*A* = 4, *B* = 3)	Total = 6 (*A* = 4, *B* = 2)
Suspect 4 (Older male)	Total = 5 (*A* = 2, *B* = 3)	Total = 4 (*A* = 2, *B* = 2)	Total = 5 (*A* = 3, *B* = 2)	Total = 6 (*A* = 3, *B* = 3)

*Decisions*: In the pre-test, *Suspect* did not have an effect on the control group’s decisions [χ^2^(1, *N* = 45) = 2.53, *p* = 0.469]. However, *Suspect* did have a significant effect on the intervention group [χ^2^(1, *N* = 45) = 8.43, *p* = 0.038]. In the post-test, *Suspect* did not have an effect on neither group’s result (control group: no false decisions; intervention group: [χ^2^(1, *N* = 45) = 2.79, *p* = 0.425]).

*Response Time*: In the pre-test, *Group* did not have a significant effect on *Response Time* [*F*(1,33) = 0.02, *p* = 0.882; η_p_^2^ < 0.01, 95% CI = [−0.30, 0.31]] nor did *Suspect* [*F*(1,33) = 1.05, *p* = 0.386; η_p_^2^ = 0.09, 95% CI = [−0.23, 0.51]]. In the post-test, *Group* had a significant effect on *Response Time* insofar as the intervention group shot faster than the control group [*F*(1,38) = 13.79, *p* < 0.001; η_p_^2^ = 0.28, 95% CI = [−0.57, −0.17]] whereas *Suspect* did not [*F*(1,38) = 1.99, *p* = 0.132; η_p_^2^ = 0.14, 95% CI = [−0.48, 0.09]].

*First Hit*: In the pre-test, *Group* did not have a significant effect on *First Hit* [*F*(1,23) = 0.15, *p* = 0.704; η_p_^2^ = 0.02, 95% CI = [−0.29, 0.59]] nor did *Suspect* [*F*(1,23) = 1.35, *p* = 0.282; η_p_^2^ = 0.15, 95% CI = [−0.69, 0.41]]. In the post-test, *Group* had a significant effect on *First Hit* insofar as the intervention group hit faster than the control group [*F*(1,31) = 16.00, *p* < 0.001; η_p_^2^ = 0.30, 95% CI = [−0.71, −0.23]] whereas *Suspect* did not [*F*(1,31) = 1.74, *p* = 0.178; η_p_^2^ = 0.10, 95% CI = [−0.50, 0.12]].

*Muzzle Position*: In the pre-test, *Group* did not have a significant effect on *Muzzle Position* [*F*(1,33) = 1.20, *p* = 0.282; η_p_^2^ = 0.02, 95% CI = [−1.03, 2.21]] nor did *Suspect* [*F*(1,33) = 0.70, *p* = 0.557; η_p_^2^ = 0.06, 95% CI = [−1.42, 2.57]]. In the post-test, *Group* did not have a significant effect on *Muzzle Position* [*F*(1,39) = 0.26, *p* = 0.613; η_p_^2^ < 0.01, 95% CI = [−1.36, 0.88]] nor did *Suspect* [*F*(1,39) = 2.44, *p* = 0.079; η_p_^2^ = 0.16, 95% CI = [−1.44, 1.50]].

Table 5 displays more detailed information of task 2 (see section 3.2). Per group and measurement time, the total number of *Shots* and the corresponding number of *Hits* result in a *Percentage*. *Ranking* indicates which of the participants’ shot hit the predefined area.

**Table 5:** Shooting characteristics progress for both groups for pre- and post-test.

	**Pre-test**		**Post-test**	
	Control group (*n* = 20)	Intervention group (*n* = 18)	Control group (*n* = 22)	Intervention group (*n* = 22)
Shots	Total = 39	Total = 38	Total = 42	Total = 52
	Mean = 1.95	Mean = 2.11	Mean = 1.91	Mean = 2.36
	(*SD* = 1.05)	(*SD* = 1.57)	(*SD* = 0.87)	(*SD* = 1.89)
Hits	Total = 31	Total = 31	Total = 34	Total = 47
	Mean = 1.55	Mean = 1.72	Mean = 1.55	Mean = 2.14
	(*SD* = 1.00)	(*SD* = 1.60)	(*SD* = 0.96)	(*SD* = 1.93)
Percentage	79.49%	81.58%	80.95%	90.38%
Ranking	None: 4 (20.00%)	None: 6 (33.34%)	None: 5 (22.72%)	None: 1 (4.54%)
	First: 14 (70.00%)	First: 8 (44.44%)	First: 11 (50.00%)	First: 19 (86.38%)
	Second: 2 (10.00%)	Second: 4 (22.22%)	Second: 4 (18.18%)	Second: 1 (4.54%)
	Third: 0	Third: 0	Third: 2 (9.10%)	Third: 1 (4.54%)

We were interested in whether both the control and the intervention group differed in these shooting characteristics in the pre- and post-test.

For *Shots*, the repeated measures ANOVA (*Time* × *Group*) showed a non-significant, small effect for the factor *Time* [*F*(1,72) = 0.04, *p* = 0.847; η_p_^2^ = 0.01, 95% CI = [−0.75, 0.75]] and a non-significant, small effect for the factor *Group* [*F*(1,72) = 0.64, *p* = 0.428; η_p_^2^ = 0.01, 95% CI = [−0.61, 0.93]]. The interaction of the factors *Time* × *Group* was non-significant with a small effect size [*F*(1,72) = 0.04, *p* = 0.839; η_p_^2^ < 0.01, 95% CI = [−0.97, 1.19]].

For *Hits*, the repeated measures ANOVA (*Time* × *Group*) showed a non-significant, small effect for the factor *Time* [*F*(1,72) = 0.22, *p* = 0.637; η_p_^2^ < 0.01, 95% CI = [−0.76, 0.76]] and a non-significant, small effect for the factor *Group* [*F*(1,72) = 1.25, *p* = 0.267; η_p_^2^ = 0.02, 95% CI = [−0.61, 0.96]]. The interaction of the factors *Time* × *Group* was non-significant with a small effect size [*F*(1,72) = 0.25, *p* = 0.619; η_p_^2^ < 0.01, 95% CI = [−0.83, 1.39]].

For *Ranking*, a two-sided Fisher-test showed that both groups did not differ significantly in the pre-test (*p* = 0.351) nor in the post-test (*p* = 0.200). The two-sided Fisher-test also showed that even within the groups there was no difference between the first and the second measurement (control group: *p* = 0.162; intervention group: *p* = 0.441).

## 4 Discussion

In the present study, we designed an individual video-based intervention training that was grounded on the 4C/ID. Inspired by the field study of Heusler and Sutter (2022), this intervention training and its tools were tested in a preregistered lab experiment. The active control group received a traditional police firearms training whereas the training of the intervention group focused on tactical gaze control, visual attention, and situational awareness. In the pre- and post-test, we measured classic marksmanship skills and the overall performance in realistic shoot/don’t shoot video scenarios.

Our results for *Hit Factor* indicate that – opposed to hypothesis 5 (and the outcome of Heusler and Sutter, 2022) – the improvement in marksmanship skills over *Time* did not depend on *Group*. Yet, the medium effect size and the narrow confidence interval are in favor of a reliable effect of *Time*. This result is interesting in that *Hit Factor* was intended as a manipulation check, i.e., it was expected that the control group, whose training was focused on the speed-accuracy trade-off, would naturally perform better or at least improve on an individual level. We believe that the high expertise of our sample may have leveled out the added value of the traditional firearms training on marksmanship skills, since the participants had an average of almost 13 years of experience as police officers (for comparison: the participants of Heusler and Sutter (2022) were second-year cadets). The effect of both trainings on *Hit Factor* does not appear to be statistically different. We cannot fully rule out that the familiarization with the gas-operated handgun caused the improvement from pre- to post-test, since only some participants were familiar with the characteristics of the gas-operated handgun utilized in our experiment (apart from the short familiarization phase in the experiment). Moreover, a part of the sample used a different kind of handgun as their service weapon. We controlled possible confounding effects, and did not find any significant effect of *Handgun* on the participants’ performance. Apparently, our participants had no difficulty in familiarizing themselves with a new (gas-operated) handgun quickly. In addition, the participants who already used the handgun on duty seemed to not benefit from their experience to a significant extent.

For *Decisions*, we hypothesized that the intervention group would improve their number of correct decisions to shoot in the shoot scenarios to a greater extent than the control group (hypothesis 3). After analysis, this hypothesis had to be rejected: In pre- and post-test, almost all participants in both groups made the correct decision. Only one participant in the intervention group refrained to shoot in the post-test (false negative). No participant made an unjustified decision (false positive) at any time. Again, we believe that the experience of our sample was decisive for this outcome as the cadets in the field study of Heusler and Sutter (2022) committed a considerable number of false positives. On the one hand, it seems that our participants were rather restrained which naturally led to low variance. On the other hand, we can only surmise whether this restraint and the associated false negatives were a cost of avoiding false positives in any case. Yet, our participants were rather willing to take damage than possibly harming innocent citizens. Although we had to reject hypothesis 3, our results reflect a great achievement by our sample. Alternative explanations for the outcomes of *Decisions*, e.g., overly obvious sequences of action in the video scenarios, are unlikely for several reasons: First, the video scenarios were designed in consultation with experts (*cf.* Heusler and Sutter, 2022). Second, the video scenarios offered high variability in terms of gender, age, and exact sequence of actions of the suspects. Third, the video scenarios were presented randomly to the participants.

The significant interaction for *Response Time* confirms our hypothesis 4: In the post-test, the intervention group did significantly shoot faster in shoot scenarios (correct positives only) than the control group, although the medium effect size and the wide confidence interval leave room for further exploration. Despite *p*-values <0.05, the main effects for *Time* and *Group* proved to be insignificant, given that the confidence interval included 0. Still, the intervention group solely was able to significantly improve from pre- to post-test (large effect size, wide CI), whereas the control group failed to do so. This is in line with the findings of Heusler and Sutter (2022). We believe that the intervention training had a considerable impact on the anticipation of the suspects’ movements, their intentions, and the threat posed to the participant. Although the control group was trimmed on speed, our results demonstrate that situational awareness and optimized gaze patterns naturally lead to quicker reactions in correctly assessed shoot scenarios.

Our outcomes for *First Hit* demonstrate that the intervention group significantly decreased the time until the first hit in the predefined areas from the pre- to post-test while the control group slightly prolonged. This finding is consequential, as it derives from the superiority of the intervention group in terms of marksmanship skills (*Hit Factor*) and reaction time (*Response Time*) as well as the newly achieved knowledge in visual attention, tactical gaze control, and situational awareness. Despite a medium-sized interaction effect, the confidence interval proved to be fairly wide again. Additionally, Figure 5 indicates that the confidence intervals for both groups’ means seem to slightly overlap in the post-test; we cannot rule out that the interaction may not be significant after all.

Furthermore, we hypothesized that the intervention group would bring their handgun up at eyesight level later before shooting than the control group (hypothesis 1). Eventually, we also had to reject this hypothesis because differences between both groups and measurement times were marginal. All participants brought their handgun up at eyesight level very close to firing a shot even before receiving their specific training. As with *Decisions*, we attribute this result to the experience of our participants.

Hypothesis 2 cannot be answered reasonably because only a fraction of the participants even closed at least one eye before firing a shot. Again, we believe that habitual behaviors that have been acquired over years of service account for this result.

With respect to the stimulus material we used, it became apparent that characteristics of the *Suspect* seemed to have had no influence on the performance of both groups. Although suspect 4 had a supposedly significant effect on *Decisions*, the statistical weakness (cell frequencies <5) opposes this finding. This result contrasts with empirical evidence regarding the effect of a suspect’s age and gender on a police officer’s use-of-force decision: On the one hand, younger suspects are more likely to experience use of force or to be shot than older suspects (Crawford and Burns, 1998; Terrill, 2007; Edwards et al., 2019). On the other hand, male suspects are much more likely to experience use of force or to be shot than female suspects (Edwards et al., 2019; Ba et al., 2021) and that even regardless of the police officer’s gender (Wright and Headley, 2020). Our results could be explained in that either experience and previous training may have eliminated possible age and gender biases or in that the participants were not susceptible to such biases in the first place. It must be acknowledged that most of the aforementioned studies originate from the US where the prevalence of fatal police encounters and private gun ownership far exceeds the German statistics.

Upon purely descriptive examination of the shooting characteristics (see Table 5), the intervention group seemed to benefit more from their training than the control group. It is noteworthy that the intervention group fired more shots in the post-test, hitting the suspect more often and, above all, earlier in correctly assessed shoot scenarios. Albeit those exploratory analyses demonstrated no statistically significant effects, there is reason to suppose that the intervention training improves marksmanship at least equivalent to traditional police firearms training – which is also substantiated by the outcomes for *Hit Factor*. Furthermore, we infer from these results that the control group’s training of marksmanship did not lead to any significant improvement.

We encouraged both groups to communicate verbally during the video scenarios. It is remarkable that almost all participants acted on this suggestion in the pre- and post-test. In the latter, most participants in the intervention group also explicitly used the unambiguous expressions suggested in the educational video.

Overall, we conclude that we were able to provide an adapted, didactically based police firearms training whose effect we have demonstrated. Our findings lead to three key insights: First, even experts seem to be susceptible to training; the participants in the intervention group improved their performance for each dependent variable from the pre- to post-test. We lacked statistical significance for *Decisions*, *Muzzle Position* and *Closed Eye(s)* which might by disappointing from a statistical point of view but demonstrates the experience and expertise of our sample: In comparison to the results of Heusler and Sutter (2022), the present participants’ performance for those variables was constantly near the performance maximum – even before receiving any training. After all, it is not surprising that experts perform better in shoot/do not decision-making scenarios than novices (Tashman et al., 2006; Ward et al., 2007; Johnson et al., 2014; Heusler and Sutter, 2020b) and show superior marksmanship (Biggs et al., 2023). But experts are also reaching their limits: Nieuwenhuys et al. (2015) showed that likewise experts were not able to overcome a robust effect of anxiety post-training. Yet, our results illustrate that our intervention training is beneficial both to novices (*cf.* Heusler and Sutter, 2022) and experts: Novices, on the one hand, demonstrate considerable learning effects in a broad range of variables, including the most crucial variable *Decisions*. Experts, on the other hand, seem to refresh those crucial learnings and elaborate the optimal execution of their correct decision (i.e., shorter response times and effective fire).

Second, the use of a gas-operated replica of a Heckler and Koch SFP9 handgun did not diminish the participants’ progress nor did it substantially decrease the level of realism; the majority of participants reported elevated stress levels at the end of the experiment. While the gas-operated handgun was more prone to malfunction than a live handgun, troubleshooting usually worked smoothly (presumably due to experience) and did not affect our statistical analyses. This insight is in line with other studies that demonstrated the efficacy of non-lethal weapons for experimental police firearms trainings (Saus et al., 2006; Oudejans, 2008; Nieuwenhuys and Oudejans, 2011; Biggs and Pettijohn, 2022).

Third, we were able to successfully implement the transition of classical classroom teaching to an educational video. In addition to the advantages mentioned in section 2.4, the participants reported approval of modern alternatives to classroom teaching. This approach is quite a standalone in that we did not find comparable studies that used educational videos to impart the necessary knowledge prior to a practical firearms training.

### 4.1 Limitations and future studies

Although one of our key insights, the use of the gas-operated handgun also represented a weakness: When firing rapidly at a high cadence, the cool gas sometimes caused the valve to freeze, resulting in malfunction (approximately with five to six participants). This disruption did primarily occur during the practical training but slowed down the whole experimental procedure. Although we solved the problem by constantly changing the magazines, other non-lethal options such as FX or paintball handguns should be considered for future studies (*cf.* Staller et al., 2017).

While participants in the field study of Heusler and Sutter (2022) took around 90 min to complete the experiment, we were able to almost halve that time despite improved input and tasks. Nevertheless, we were not able to run a retention test with our participants in order to evaluate the sustainability of our effects. In addition, Heusler and Sutter (2022) already suggested to include a third group omitting the pre-test. Given our sample size, we could not comply with this recommendation, hence conceivable learning effects of the pre-test can still not be ruled out. In a follow-up study, we might also add further tests to distinguish between mere refreshment and new learning. A more sophisticated evaluation of the participants’ level of knowledge pre- and post-training could yield valuable insights on potential for improving the theoretical content.

For some effects, the somewhat wide confidence intervals indicate a degree of uncertainty regarding the true effect. For *First Hit*, this finding is supplemented by Figure 5. In a follow-up study, a larger sample is ought to shed light on this.

Compared to Heusler and Sutter (2022), we extended the video scenarios with three additional persons; yet, more variance in age, gender, ethnicity, and general appearance would yield a more realistic setting. It is a well-established finding that a counterpart’s appearance and attributed ethnicity can significantly deteriorate the outcome in police-civilian interaction (Ross, 2015; Worrall et al., 2018; Edwards et al., 2019; Wright and Headley, 2020; Ba et al., 2021). Additionally, in our video scenarios we used a weapon that was clearly identifiable as a handgun and therefore left no room for hesitation or vagueness. To increase the level of realism and to soften the correct-decision-wrong-decision scheme, further video scenarios could contain rather ambiguous objects that are potentially but not necessarily or imminently hazardous (e.g., glass bottles, knives, pepper spray, etc.). A higher degree of ambiguity would also increase variance among experts and might reveal possible group differences.

Within this framework, different approaches could elevate the quality of police firearms training: Elaborate roleplays would maximize realism whereas augmented/virtual reality solutions are a more sophisticated way to simulate various police operations (*cf.* Tawa et al., 2022). Eventually, the task itself could be adapted in a way that no information is given at the beginning. Thus, the participants would not be primed about the use of their service weapon but rather had to make their decisions isolated from external information and under more realistic circumstances. Situations that require the use of the service weapon will always result in high stress levels and anxiety for even the most experienced police officers. Therefore, training under maximized levels of stress and anxiety will optimally prepare police officers for real-life encounters (Oudejans, 2008; Nieuwenhuys and Oudejans, 2011; Liu et al., 2018). To additionally elevate the levels of stress and anxiety (especially when using non-lethal weapons), the participants could be informed about being shot at by the suspect if they reacted too slow (*cf.* Nieuwenhuys and Oudejans, 2010). A corresponding experimental setup and equipping the participants with protective clothing should induce stress and anxiety, regardless of whether shots were actually being fired back or not. Given this setting, an extension of the theoretical and practical training on methods of stress reduction and coping with anxiety would be advisable.

Ultimately, the absence of false positive decisions in the pre- and post-test raises the question of whether our intervention training might have had negative side-effects in don’t shoot situations, which we were unable to capture in the present experiment as our sample’s proficiency and expertise are not representative of most police officers. In order to increase the validity and reliability of the training, the experiment should be conducted again on a less experienced sample (such as first-year police officers). Optionally, assessing the training’s suitability for the challenges of special forces seems feasible. Furthermore, most of our variables only focused on the shoot scenarios which is why future studies might find ways to operationalize measurements that compare the relation of false negatives and false positives more thoroughly.

## 5 Conclusion

To address the real life demands, police officers must optimally be prepared by constantly shifting toward a more sophisticated firearms training. Our intervention training that focused on situational awareness, tactical gaze control, and visual attention also enabled senior police officers to react quickly and adequately in realistic shoot/don’t shoot scenarios. In our experiment, an individual video-based training format improved the participants’ performance more than traditional police firearms training. We conclude that the intervention training is superior to the traditional police firearms training not only for cadets, but also experienced officers will benefit from such an approach. In times of social tensions, good policing is an indispensable asset. Although police firearms training only represents a fraction of police work, the impact on social peace and cohesion should not be underestimated. Therefore, it is in the best interest of a law enforcement agency that all officers equipped with service weapons demonstrate a high degree of proficiency. Hence, police officers should regularly review their theoretical knowledge and practical skills to a degree that exceeds bullseye shooting.

Regarding the theoretical framework, we infer that the 4C/ID served as a useful fundament for the intervention training. Although the model is not intended to unfold its full impact in a short lab experiment, our results suggest that a more comprehensive police firearms curriculum could be based on the 4C/ID. To achieve even better outcomes, this curriculum might include the traditional police firearms training extended by approaches that emphasize the importance of situational awareness, tactical gaze control, and visual attention. Training with video-based scenarios and educational videos has also proven to be an economical solution for an advanced police firearms training. A multi-method approach that incorporates classical teaching methods and modern, digital options will eventually enrich the didactic fundament as well as technology-driven innovations related to augmented/virtual reality. Future research and training concepts should ensure that police officers are given the best possible prerequisites to correctly assess situations, distinguish hazardous from non-hazardous objects, and make an accurate decision.

## Data availability statement

The raw data supporting the conclusions of this article will be made available by the authors, without undue reservation.

## Ethics statement

The studies involving humans were approved by the Ethics Committee of the German Police University (“Ethik Kommission der Deutschen Hochschule der Polizei”) (approval number: “DHPol-EthK.2022.Olm3”). The studies were conducted in accordance with the local legislation and institutional requirements. The participants provided their written informed consent to participate in this study. The individual(s) provided their written informed consent for the publication of any identifiable images or data presented in this article.

## Author contributions

JO: Writing – original draft, Writing – review & editing. CS: Writing – review & editing, Writing – original draft. SS: Writing – review & editing.

## Footnotes

^1^Exceptions: In shoot scenario A, the older man did not telephone and retrieved the handgun from his jacket pocket both times. In shoot scenario B, the older woman retrieved the handgun from her back pocket both times.
